# Use of a Computerized C-Reactive Protein (CRP) Based Sepsis Evaluation in Very Low Birth Weight (VLBW) Infants: A Five-Year Experience

**DOI:** 10.1371/journal.pone.0078602

**Published:** 2013-11-11

**Authors:** Sarah A. Coggins, James L. Wynn, Melissa L. Hill, James C. Slaughter, Asli Ozdas-Weitkamp, Osman Jalloh, L. Russell Waitman, Randy J. Carnevale, Jörn-Hendrik Weitkamp

**Affiliations:** 1 School of Medicine, Vanderbilt University, Nashville, Tennessee, United States of America; 2 Department of Pediatrics, Monroe J. Carell Children's Hospital at Vanderbilt, Vanderbilt University, Nashville, Tennessee, United States of America; 3 Department of Biostatistics, Vanderbilt University, Nashville, Tennessee, United States of America; 4 Department of Biomedical Informatics, Vanderbilt University, Nashville, Tennessee, United States of America; Emory University School of Medicine, United States of America

## Abstract

**Background:**

Serial C-reactive protein (CRP) values may be useful for decision-making regarding duration of antibiotics in neonates. However, established standard of practice for its use in preterm very low birth weight (<1500 g, VLBW) infants are lacking.

**Objective:**

Evaluate compliance with a CRP-guided computerized decision support (CDS) algorithm and compare characteristics and outcomes of compliant versus non-compliant cases. Measure correlation between CRPs and white blood count (WBC) indices.

**Methods:**

We examined 3 populations: 1) all preterm VLBW infants born at Vanderbilt 2006–2011 – we assessed provider compliance with CDS algorithm and measured relevant outcomes; 2) all patients with positive blood culture results admitted to the Vanderbilt NICU 2006–2012 – we tested the correlation between CRP and WBC results within 7 days of blood culture phlebotomy; 3) 1,000 randomly selected patients out of the 7,062 patients admitted to the NICU 2006–2012 – we correlated time-associated CRP values and absolute neutrophil counts.

**Results:**

Of 636 VLBW infants in cohort 1), 569 (89%) received empiric antibiotics for suspected early-onset sepsis. In 409 infants (72%) the CDS algorithm was followed; antibiotics were discontinued ≤48 hours in 311 (55%) with normal serial CRPs and continued in 98 (17%) with positive CRPs, resulting in significant reduction in antibiotic exposure (p<0.001) without increase in complications or subsequent infections. One hundred sixty (28%) were considered non-compliant because antibiotics were continued beyond 48 hours despite negative serial CRPs and blood cultures. Serial CRPs remained negative in 38 (12%) of 308 blood culture-positive infants from cohort 2, but only 4 patients had clinically probable sepsis with single organisms and no immunodeficiency besides extreme prematurity. Leukopenia of any cell type was not linked with CRPs in cohorts 2 and 3.

**Conclusions:**

CDS/CRP-guided antibiotic use is safe and effective in culture-negative VLBW infants. CRP results are not affected by low WBC indices.

## Introduction

In the NICU, “suspect sepsis” is by far the most common diagnosis and antibiotics are the most frequently prescribed medications [1]. Low positive predictive value for widely available diagnostic testing methods (such as the white blood count [WBC] and C-reactive protein [CRP]) in this population, in conjunction with the increased risk of sepsis-related mortality once symptomatic [2], places significant diagnostic value on the clinical assessment and supports initiation of early empiric antimicrobials in at-risk or symptomatic preterm infants. Based on the current recommendations to treat stable NICU patients with antibiotics for 48–72 hours with negative blood culture results and 7–14 days for blood-culture positive or clinical probable infection, between 11 and 23 uninfected infants are treated for every case of documented sepsis [3]–[5]. Potential adverse effects of unnecessary antibiotic usage include short-term (e.g. pain, infection, infiltration) and long-term complications (e.g. necrotizing enterocolitis (NEC), hearing impairment, antimicrobial resistance development) [6]–[9]. Some studies suggest that serial CRP measurements (in combination with WBC) may be a useful tool to help the experienced clinician in decision-making regarding initiation and duration of antibiotics in stable NICU patients [10], [11]. Using a clinical pathway for neonatal sepsis, which is based primarily on CRP determinations, may minimize antibiotic exposure and shorten hospital stays in asymptomatic infants [12]. However, there is no established standard of practice for the use of CRP in very low birth weight (<1500 g, VLBW) preterm infants. We developed and instituted a CRP-based protocol to address the clinical dilemma “to treat or not to treat” for infants without a positive blood culture within 48 hours [13].

**Figure 1:**
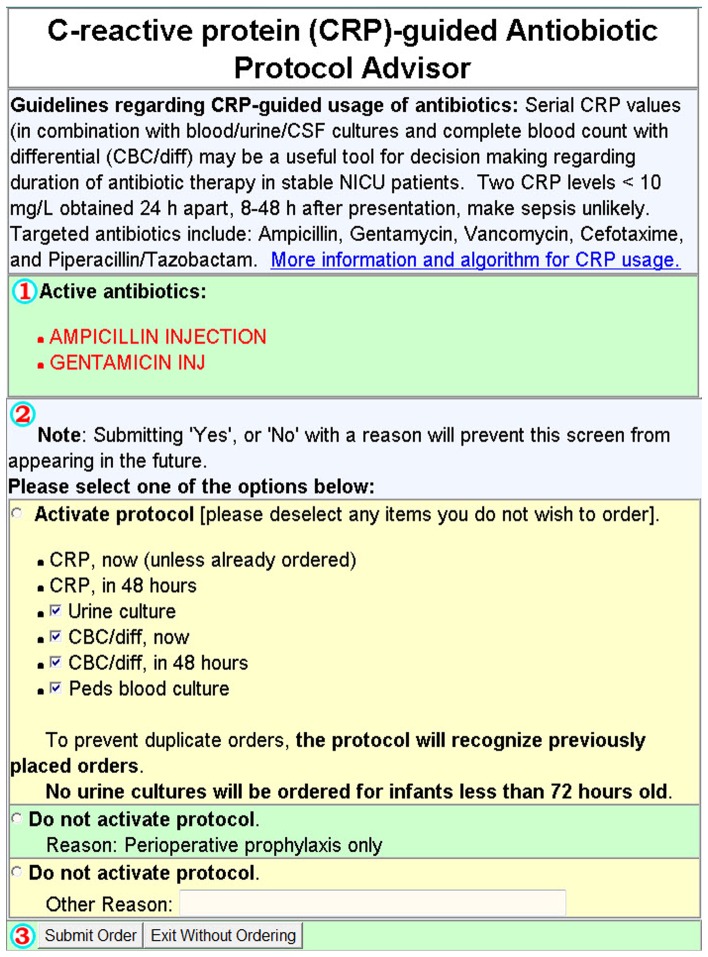
Screenshot CRP protocol. Entry of an antibiotic order into the computerized system produced this screen, prompting the initiation of the CRP protocol. The protocol included automated orders for a blood culture, CRP, and WBC with differential at admission, followed by a repeat CRP and WBC with differential 48

**Figure 2:**
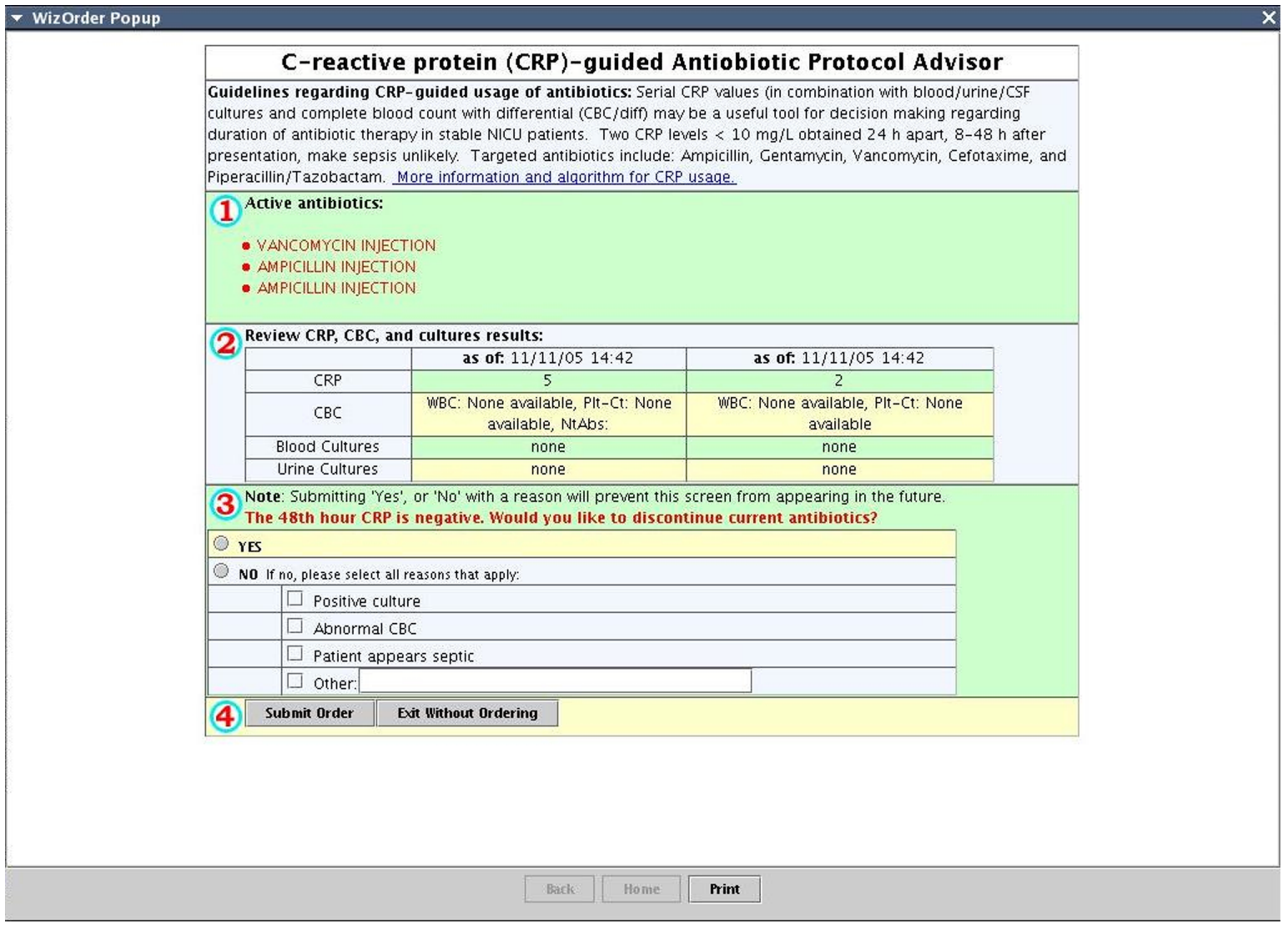
Screenshot decision support to stop antibiotics. The CRP protocol's recommendation on antibiotic use was based on repeat CRP and WBC results drawn 48 hours following initial evaluation. In cases with two consecutively negative CRP results (CRP <10 mg/L), this discontinuation screen appeared, allowing the provider to choose to stop antibiotics. If the provider chose to continue antibiotics despite negative CRP results, they were prompted to provide reasoning for doing so. No complete white blood count (CBC) data is depicted in this screenshot, because a “test” patient had to be generated to produce this figure.

Computerized physician order entry (CPOE) with clinical decision support (CDS) in the NICU setting has been shown to decrease variance in medication administration, improve protocol compliance and reduce mortality [14]–[17]. We implemented the CRP-protocol as a CDS module integrated into our institution's CPOE system. Entry of an antibiotic order into the CPOE system triggered the initiation of the CRP-protocol. The initiation screen of the CDS module recommended ordering a blood culture, CRP, WBC with differential at admission followed by a repeat CRP and WBC with differential 48 hours later for surveillance purposes (Figure 1). The recommendation for further antibiotic use was based on 2 consecutive CRP results within 48 hours of the initiation of antibiotic orders and blood culture results. Two consecutive negative CRP results (CRP <10 mg/L) triggered the discontinuation screen where the CDS module displayed evaluating the option for discontinuing antibiotic orders in case of negative blood cultures (Figure 2).

Traditionally, WBC indices are used as diagnostic sepsis markers in early- and late-onset sepsis [18]. Therefore, WBC data obtained simultaneously with CRP values were displayed with blood culture results to the provider as part of the discontinuation screen. To our knowledge, there have been no published reports assessing the correlation between WBC indices and CRP values in VLBW infants. The purpose of this study was to evaluate the compliance, effectiveness and safety of implementing CRP-based guidelines in determining empirical antibiotic use in suspected early-onset sepsis in preterm VLBW infants and to measure the association between WBC indices and CRP values over time.

## Methods

### Patients

This study has been approved by the Vanderbilt University Institutional Review Board (IRB #120953 and 111600). Consent by the next of kin, caretakers, or guardians on the behalf of the infant participants, for their stored medical records used for research, was specifically waived by the approving IRB. We performed a retrospective chart review on 3 separate sets of patients:

Cohort 1: All inborn preterm infants <1500 g admitted at Vanderbilt between January 2006 and December 2011 with negative blood cultures during the first week post-partum. We excluded patients with congenital anomalies and/or surgery performed within the first 3 postnatal days typically resulting in prolonged empiric antibiotic use. We compared the number of antibiotic doses, days on antibiotics, as well as specific morbidities affecting VLBW infants (CLD [supplemental oxygen requirement ≥36 weeks postconceptional age], necrotizing enterocolitis [NEC, Bell Stage ≥IIa, intraventricular hemorrhage [IVH], surgical ligation of patent ductus arteriosus [PDA], secondary infection or meningitis). Our primary outcome designated *a priori* was the length of antibiotic treatment of infants with negative blood cultures who were treated based on 2 consecutive CRP values obtained at initial evaluation (t0) and at 48 hours (t48) (“CRP protocol compliant”) and those that were treated outside the protocol (“CRP protocol non-compliant”). Patients for whom antibiotics were never started were excluded from the study (n = 67).

Cohort 2: All patients admitted to the Vanderbilt NICU between January 1, 2006, and June 20, 2012 with positive blood cultures. We tested the correlation between CRP, WBC (absolute neutrophils, lymphocytes, monocytes, and eosinophils), and blood culture results within 7 days of diagnostic phlebotomy. We excluded non-viable infants or infants who did not survive past the first 48 hours post-partum, patients no longer hospitalized at Vanderbilt 48 hours after birth, and patients who did not have at least one time-correlated set of CRP and neutrophil data. We stratified CRP and WBC correlations by positive blood cultures with and without coagulase-negative *Staphylococcus* (CONS), and cases with and without documented clinical signs of sepsis (clinically overt sepsis as defined by Fanaroff *et al.* [19]. Specific parameters included significant change in vital signs such as hypothermia (<36°C rectal) or fever (>38°C rectal), increased severity and frequency of apnea/bradycardia events, new or increased pulmonary or cardiovascular support, and new feeding intolerance).

Cohort 3: 1,000 randomly selected patients out of the 7,062 patients admitted to the NICU during the same study period as in cohort 2. We correlated all time-associated CRP values and absolute neutrophil counts that were accessible in the Vanderbilt electronic medical records. The same exclusion criteria as in cohort 2 applied.

### Definitions

Confirmed positive blood cultures were defined as any single culture positive for non-contaminant, non-CONS bacteria or for *Candida* species. CONS infections were considered confirmed if at least 1 repeat positive culture was present within 72 hours of the initial positive blood culture. A positive CRP result was defined as any value greater than 10 mg/L [12]. For the assessment of the CRP-based CDS protocol in the first week of life in VLBW infants, we defined a normal total white blood cell count as 5–20×10^3^ cells/μL and a normal total neutrophil count as >2,500/μL [20]. To evaluate the impact of neutropenia, lymphopenia, monopenia, and eosinopenia on CRP as a sepsis marker in bacteremic NICU patients, we defined applied absolute counts of <3,500/µl, <2,000/µl, <250/µl, and <150/µl, respectively [21]–[25].

### Clinical Decision Support (CDS) Protocol

The CRP protocol included automated orders for a blood culture, CRP, WBC with differential at time of antibiotic order followed by a repeat CRP and WBC with differential 48 hours later (Figure 1). If the blood culture and both CRP values remained negative (CRP <10 mg/L) at 48 hours, the CDS protocol would recommend stopping antibiotics (Figure 2). WBC results were also displayed at the time of decision support. Providers were prompted to indicate reasons for continuing antibiotics despite negative serial CRP values.

### Analytical methods

Continuous variables were described using the median, 25th and 75th percentiles, and categorical variables were described using percentages. The Wilcoxon signed rank test (2 group) or Kruskal-Wallis test (3 groups) were used to determine if the continuous variables differed between compliance groups. Pearson's Chi-squared test was used to evaluate differences by compliance group for categorical variables. Separate multivariable logistic regression were fit to determine if the binary outcomes of death, NEC, PDA, CLD or IVH were associated with compliance group while controlling for gestational age, ventilator days and nasal CPAP days. To evaluate the association between the absolute neutrophil count (ANC) and CRP, we used a linear mixed effects regression model. This regression model included a random intercept term to control for taking repeated observations on subjects over time.

## Results

### Compliance with the computerized protocol (patient cohort 1)

We identified 636 culture-negative VLBWs within the 5-year study period of which 569 (89%) received empiric antibiotics in the first week post-partum for possible early-onset sepsis. Antibiotics were discontinued within 48 hours in 311 infants (55%) with negative CRP results and continued in 98 infants (15%) for at least one CRP value >10 mg/L, totaling 409 infants deemed “CRP-protocol compliant” (Comp). Overall compliance with the computerized CRP protocol was 72%, as empiric antibiotics were continued >48 hours in the setting of a negative blood culture with negative serial CRP results in 160 patients (28%). We designated these 160 cases as “CRP-protocol non-compliant” (Non-Comp). Patients in the Non-Comp group were of lower gestational age and birth weight compared to the Comp group (Table 1). The Non-Comp group had more days on the ventilator or on nasal CPAP and received oxygen longer (Table 1). Among the reasons given by providers for continuing antibiotics in the Non-Comp group were: abnormal WBC indices, preterm premature rupture of membranes (PPROM), foul smelling amniotic fluid, maternal chorioamnionitis, maternal fever, thrombocytopenia, twin's clinical status (elevated CRP), and abdominal distension (Table 2).

**Table 1:** Patient demographics by CRP-protocol compliance status.^1^

	Comp (N = 409)	Non-Comp (N = 160)	p-value^2^
Gestational age	29 (27; 30)^3^	27 (25; 29)	<0.001^4^
Birth weight (g)	1130 (870; 1320)	870 (680; 1088)	<0.001^4^
Male (%)	54	53	0.8^5^
Race (%)^6^	C62/AA27/H7/O2/U3	C56/AA32/H9/O1/U1	0.24^5^
Ventilator days	2 (0; 7)	6 (1; 22)	<0.001^4^
Nasal CPAP days	1 (0; 4)	1 (0; 7.2)	0.014^4^
Oxygen days	10 (2;002042)	44 (17; 85)	<0.001^4^
WBC (x10^3^/µL)	7.5 (5.1; 10.4)	8.3 (4.9; 14.8)	0.15^4^
ANC (cells/µL) t0^7^	2,270 (1,309; 3,938)	2,010 (1,089; 7,294)	0.98^4^
ANC t0 low^8^ (%)	54	59	<0.001^5^
ANC (cells/µL) t48^9^	3,669 (2,381; 5,759)	3,866 (1,506; 9,808)	0.83^4^
ANC t48 low (%)	26	35	<0.001^2^

1 Compliance with the CRP-protocol in the setting of negative blood cultures was defined as (1) antibiotics were discontinued ≤48 hours after 2 consecutive negative CRP results, or continued if at least one positive CRP was present [“CRP-protocol compliant” (Comp)], and (2) antibiotics were continued >48 hours despite negative serial CRP results [“CRP-protocol non-compliant” (Non-Comp)].

2 Pair-wise comparison between Comp and Non-Comp group.

3 Median (lower quartile; upper quartile).

4 Wilcoxon test.

5 Pearson test.

6 C =  Caucasian, AA  =  African American, H =  Hispanic, O =  Other, U =  Unknown.

7 Absolute neutrophil count at time of initial sepsis evaluation.

8 <2,500/µL.

9 Absolute neutrophil count at 48 hours.

**Table 2:** Reasons stated by clinical providers in the clinical decision support (CDS) module for continued antibiotic therapy despite negative serial CRP values.

Provider reason^1^	Number of infants (%)
Decreased total white blood counts (WBC)	44 (30.6)
Maternal history^2^	28 (19.4)
Neutropenia	25 (17.3)
Increased WBC	23 (16.0)
No reason given	18 (12.5)
Change in CRP but ≤10 mg/L	14 (9.7)
Thrombocytopenia	14 (9.7)
Clinical status of the infant^3^	12 (8.6)
Twin's clinical status	8 (5.6)

1 Providers were able to state more than one reason.

2 Included preterm premature rupture of membranes (PPROM), Group B *streptococcus* (GBS) status, chorioamnionitis, fever.

3 Included concerns for respiratory and gastrointestinal pathology.

**Figure 3:**
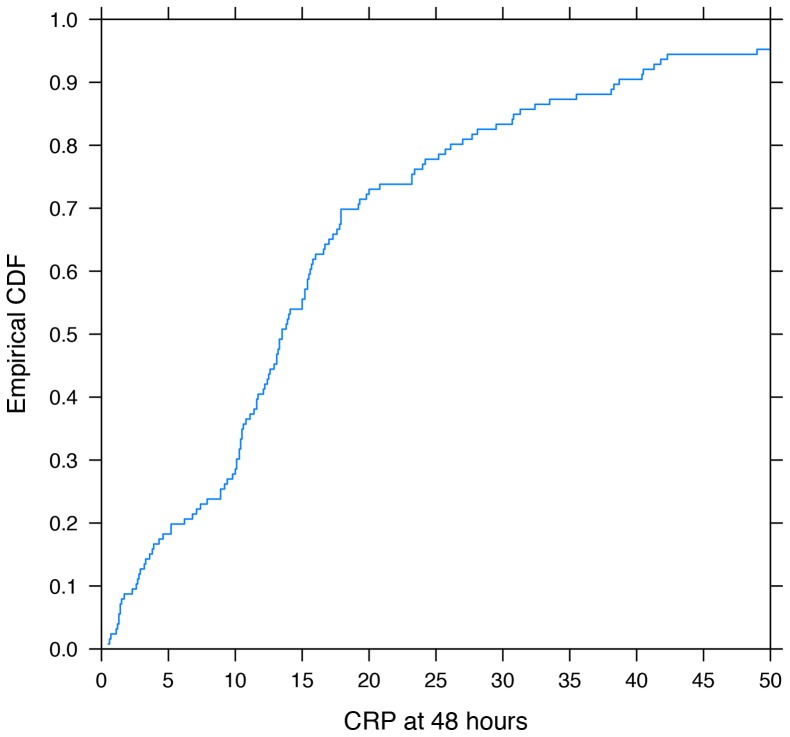
Cumulative distribution function (CDF) plot of t_48_ CRP values in infants with continued antibiotics for elevated CRP value (s). The blue line depicts the distribution of CRP values among 126 infants in which antibiotics were continued because of elevated t0 and/or t48 CRP results; 44.5% had t48 CRP values 10–20 mg/L and 34.4% had values >20 mg/L.

Of all infants in whom antibiotics were continued because of elevated CRP results on admission, only 32% had CRP values >10 mg/L at 48 hours. Of all 126 infants with continued antibiotics because of positive CRP values at either t0 or t48, at 48 hours, 27% had values between 10 and 15 mg/L, 17.5% between 15 and 20 mg/L, 17.6% between 20 and 40 mg/L and 16.8% >40 mg/L (Figure 3).

### Impact of stopping empiric antibiotics based on the computerized CRP protocol (patient cohort 1)

The 409 infants in the Comp group received significantly less doses of ampicillin (5 versus 14, p<0.001) and a smaller total dose of ampicillin (0.52 mg/g versus 1.38 mg/g, p<0.001) compared to the 160 patients in the Non-Comp group (Table 3). Within the Comp group, 15 (4.8%) were committed to a full course of antibiotics after the initial work-up but within the first 10 days post-partum. Nine infants had CONS-positive blood cultures, 5 developed NEC and 1 had non-CONS-positive blood cultures. The Comp group had no increase in total prematurity-associated morbidities compared to the Non-Comp group (Table 4). The incidence for surgical PDA, CLD or subsequent positive blood cultures with CONS were significantly lower in the Comp group compared to the Non-Comp group (p<0.004). Patients who had their antibiotics discontinued had a lower incidence of NEC and NEC-attributable death (Table S1). However, antibiotic continuation was associated with a reduced incidence for early NEC. The study was underpowered to test the effect of protocol compliance on timing of NEC. The incidence of all other investigated outcomes was similar, except a significantly reduced incidence in death (p = 0.003) and IVH (p = 0.011) in the Comp group. After multivariable analysis adjusting for gestational age, ventilator and nasal CPAP days, however, there were no differences in outcomes between the groups.

**Table 3:** Ampicillin usage by CRP-protocol compliance status.^1^

	Comp (N = 409)	Non-Comp (N = 160)	P-value^2^
Number of doses	5 (5; 7) 3	14 (12; 15)	<0.001
Numbers of hours on Ampicillin	56 (51; 71)	159 (142; 170)	<0.001
Milligram Ampicillin/gram BW^4^	0.52 (0.49; 0.65)	1.38 (1.24; 1.48)	<0.001

1 Compliance with the CRP-protocol in the setting of negative blood cultures was defined as (1) antibiotics were discontinued ≤48 hours after 2 consecutive negative CRP results or continued after at least one positive CRP value [“CRP-protocol compliant” (Comp)] and (2) antibiotics were continued >48 hours despite negative serial CRP results [“CRP-protocol non-compliant” (Non-Comp)].

2 Kruskal-Wallis test.

3 Median (lower quartile; upper quartile).

4 BW  =  birth weight.

**Table 4:** Outcomes (%) by CRP-protocol compliance status.^1^

	Comp (N = 409)	Non-Comp (N = 160)	P-value^2^
Necrotizing enterocolitis (Bell ≥ IIa)	8	12	0.065
Death	6	14	0.003
Chronic lung disease^3^	26	52	<0.001
Intraventricular hemorrhage	31	42	0.011
Persistent ductus arteriosus ligation	7	17	<0.001
Bacterial sepsis	12	20	0.011
CONS^4^ sepsis	12	22	0.004
Fungal sepsis	1	1	0.553
Any NEC, death, or bacterial sepsis	21	36	<0.001

1 Compliance with the CRP-protocol in the setting of negative blood cultures was defined as (1) antibiotics were discontinued ≤48 hours after 2 consecutive negative CRP results or continued after at least one positive CRP value [“CRP-protocol compliant” (Comp)], and (2) antibiotics were continued >48 hours despite negative serial CRP results [“CRP-protocol non-compliant” (Non-Comp)].

2 Pearson test.

3 Defined as supplemental oxygen requirement ≥36 weeks postconceptional age.

4 CONS  =  Coagulase-negative *Staphylococcus*.

### Association between CRP and WBC indices within the computerized protocol (patient cohort 1)

Among the 311 VLBW infants where antibiotics were stopped within 48 hours, 204 infants (66%) had abnormal WBC indices. Among these 204 infants, over 80% had low WBC indices and 20% had high WBC indices (as defined in Methods). In over 80% of these 204 cases, neutrophil counts recovered to values >2,500/μL at least once during the 48 hour diagnostic period. Providers elected to continue antibiotics despite negative cultures and negative serial CRP values in 160 infants for reasons shown in Table 2. Abnormal WBC indices was the most common indicated reason for prolonging antibiotic use despite negative serial CRP values and included leukopenia (31%), neutropenia (17%), and leukocytosis (16%).

### Association between CRP and WBC indices in neonates with positive blood cultures (patient cohort 2)

**Figure 4:**
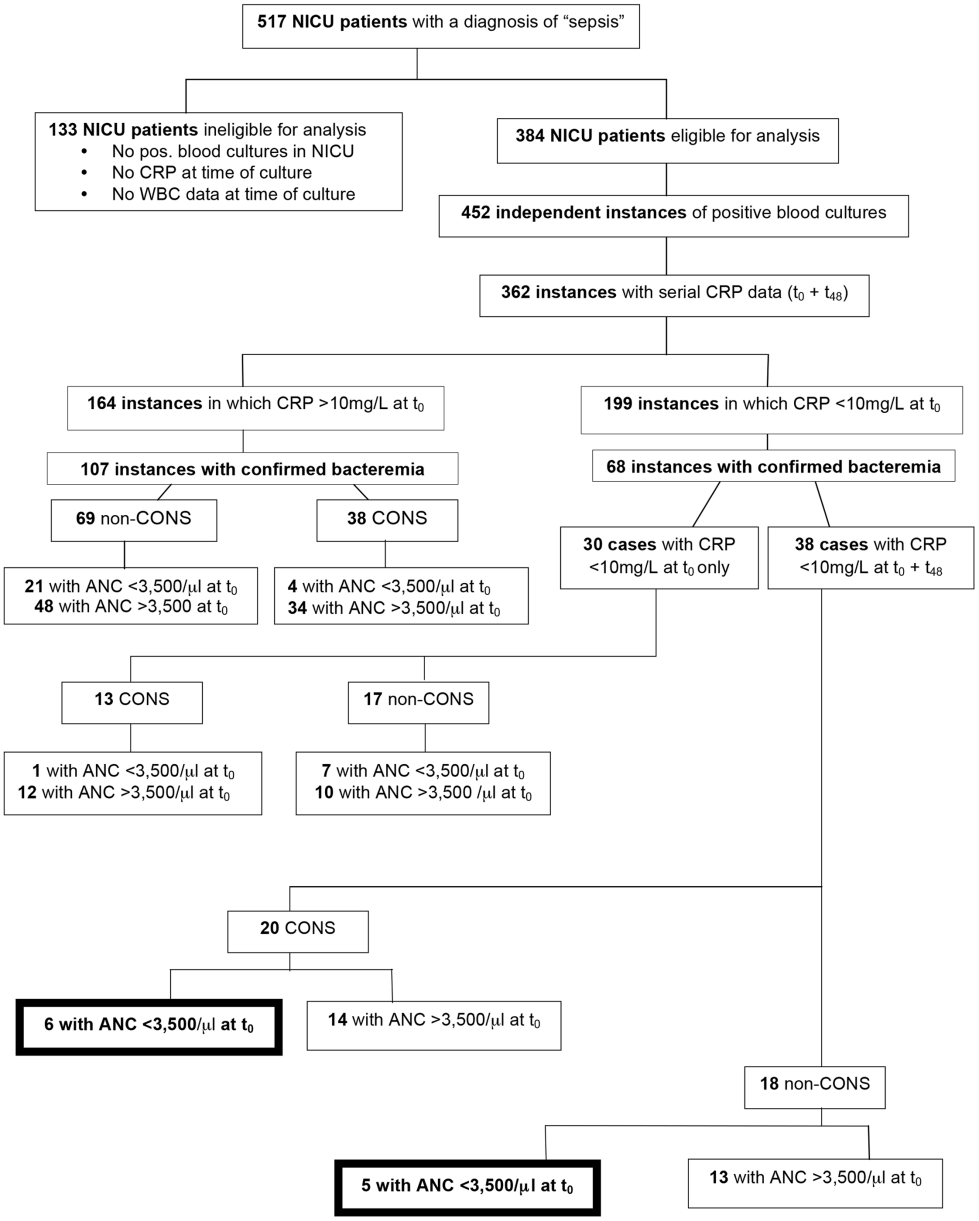
Flow diagram detailing analysis of serial CRP values and ANCs in blood culture positive neonates. Of 517 patients available for analysis during the study period, 384 fit the inclusion/exclusion criteria. Among these eligible patients, we analyzed 362 independent cases of positive blood cultures with available ANC and serial CRP data. These cases were stratified according to initial (t_0_) CRP status, serial (t_48_) CRP status, organism class (CONS vs. non-CONS), and finally by ANC status at initial (t_0_) evaluation. Only cases deemed to be confirmed positive blood cultures were included in this stratification scheme.

Outside anecdotal reports, we are not aware of published systematic evaluations of the impact of abnormal WBC indices on CRP rise with culture-proven sepsis. Therefore we tested the consequence of neutropenia, lymphopenia, monopenia and eosinopenia on CRP elevation in bacteremic NICU patients. We identified 362 independent cases of positive blood cultures with available serial CRP data in 308 individual NICU patients between January 1, 2006, and June 20, 2012 and analyzed associated WBC indices and CRP results (Figure 4). The mean (range) gestational age was 29.0 (22–42) weeks with a mean (range) birth weight of 1,405 (264–4,766) grams. Race and sex distributions in this cohort were similar to the cohort with negative blood cultures shown in Table 1.

At initial evaluation, 199 of the 362 (54.8%) cases of reported positive blood cultures had negative (<10 mg/L) CRP values, of which 68 (33%) were deemed to be confirmed positive blood cultures as defined above. The distribution of gram-negative and gram-positive bacteria in patients with negative or positive CRP results at initial evaluation was similar, indicating the relatively low sensitivity of initially negative CRP results (Table 5).

**Table 5:** Organisms isolated from positive blood cultures in patients with confirmed infections and CRP <10 mg/L (N = 66) or >10 mg/L (N = 109) at initial evaluation.^1^

Organism (N)	CRP <10 mg/L	CRP >10 mg/L
Gram-negative bacteria	*Acinetobacter baumanii* (1)	*Acinetobacter* ssp. (1)
		*Citrobacter freundii* (1)
		*Delftia acidovorans* (1)
	*Enterobacter cloacae* (1)	
	*Escherichia coli* (9)	*Escherichia coli* (4)
		*Klebsiella oxytoca* (2)
	*Klebsiella pneumoniae* (3)	*Klebsiella pneumoniae* (11)
	*Morganella morganii* (1)	*Morganella morganii* (1)
		*Neisseria polysaccharea* (1)
		*Serratia marcescens* (3)
Gram-positive bacteria	*Enterococcus faecalis* (12)	*Enterococcus faecalis* (4)
		*Enterococcus faecium* (1)
		*Lactobacillus acidophilus* (1)
	*Staphylococcus aureus* (3)	*Staphylococcus aureus* (MRSA) (4)
		*Staphylococccus aureus* (MSSA) (6)
	*Staphylococcus* coag.-neg. (33)	*Staphylococcus* coag.-neg. (38)
	*Streptococcus agalactiae* (1)	*Streptococcus agalactiae* (6)
	*Streptococcus alpha* (1)	
		*Streptococcus gamma* (2)
Fungi	*Candida albicans* (3)	*Candida albicans* (2)
		*Candida krusei* (1)
	*Candida parapsilosis* (1)	*Candida parapsilosis* (2)

1 Some patients had more than one organism present on initial culture.

In 38 (12%) infants of 308 with confirmed positive blood cultures, serial CRP values remained negative. A list of 39 organisms identified from 38 patients is presented in Table 6. Twenty cases were cultures positive for CONS, a condition with controversial clinical significance and previously associated with low sensitivity of CRP [12], [26]. Therefore, we performed a detailed chart review on the 18 infants with confirmed non-CONS cultures and negative serial CRP values. In this group 6 patients had mixed cultures and were not septic appearing. Four patients had congenital or hereditary conditions consistent with a poor immune response (e.g. DiGeorge Syndrome with SCID phenotype, congenital L-carnitine deficiency [27], [28]). Only 4 more patients had clinically significant infections, however none were associated with culture-proven meningitis or resulted in death. All of these 4 patients were either ELBW (n = 3) or VLBW (n = 1), supporting published data on lower sensitivity of CRP in the extreme premature population [29], [30].

**Table 6:** Organisms isolated from positive blood cultures in patients with confirmed infections (N = 38) and CRP persistently <10 mg/L.

Category	Organism (N)
Gram-negative bacteria	*Acinetobacter baumanii* (1)
	*Escherichia coli* (5)
	*Klebsiella pneumoniae* (1)
Gram-positive bacteria	*Enterococcus faecalis* (6)
	*Staphylococcus aureus* (13)
	*Staphylococcus* coagulase-negative (20)
	*Streptococcus agalactiae* (1)
	*Streptococcus alpha* (1)
Fungi	*Candida albicans* (1)

To test the hypothesis that false-negative CRP results are more commonly observed in cases of abnormal WBC indices, we compared the frequency of low WBC values between cases with confirmed positive blood cultures and either positive or negative serial CRP results (Table 7). In 11 of these 38 cases (29%) with confirmed positive blood cultures and persistently negative CRP results, infants had neutrophil counts of <3,500/μL. Six of those 11 cases had positive blood cultures with CONS. In all cases of non-CONS-positive blood cultures, neither neutropenia, lymphopenia, monopenia nor eosinopenia were more commonly observed in patients with negative compared to positive CRP results. When we analyzed the data from 68 patients with proven positive blood cultures (non-CONS), we found no statistically significant correlations between WBC indices and CRP (p>0.08).

**Table 7:** Number of cases (N) with confirmed positive blood cultures and low WBC indices with positive (>10 mg/L) CRP values versus negative (<10 mg/L) serial CRP results.^1^

	Neutropenia (<3,500/µl)		Lymphopenia (<2,000/µl)		Monopenia (<250/µl)		Eosinopenia (<150/µl)	
Serial CRP	Pos.	Neg.	Pos.	Neg.	Pos.	Neg.	Pos.	Neg.
CONS^2,3^, N (%)	5 (11.6)	6 (35.3)	11 (25.6)	1 (5.9)	2 (4.7)	0 (0)	12 (27.9)	2 (11.8)
Non-CONS^4^, N (%)	17 (31.5)	5 (31.2)	23 (42.6)	2 (12.5)	6 (11.1)	0 (0)	28 (51.9)	5 (31.2)

1 =  Only cases with a full set of reported WBC and differential were analyzed.

2 =  CONS  =  coagulase-negative *Staphylococcus*.

3 =  Total serial CRP pos. N = 43; total serial CRP neg. N = 17.

4 =  Total serial CRP pos. N = 54; total serial CRP neg. N = 16.

To determine if the frequency of false-negative CRP results is higher in neutropenic patients compared to infants with normal neutrophil counts, we reviewed 27 cases of persistently negative CRP values despite confirmed positive blood cultures in NICU patients with ANCs >3,500/μL. We found 3 non-CONS cases with clinically probable sepsis as defined in Ref. 19, indicating a similar yet overall very rare incidence of false-negative CRP results in non-neutropenic infants.

### Associations between CRP and WBC indices within the general NICU population (patient cohort 3)

False negative CRP results in proven clinically significant sepsis have occurred in association with neutropenia [10], [12]. To determine if a possible relationship exists between CRP results and absolute neutrophil counts, we analyzed 3,113 time-correlated sets of CRP and ANC values from 1,000 random NICU patients (with and without positive blood cultures) and detected a weak but highly statistically significant positive correlation (R = 0.25, p<0.0001).

## Discussion

The implementation of a computerized CRP-based early sepsis evaluation protocol for VLBW infants was associated with a reduction of antimicrobial days/doses without evidence of increased risk in infants treated per the algorithm. Clinician compliance with the protocol was reasonable (72%) and subsequent suspect sepsis evaluations were infrequent in infants following the week of CRP protocol-guided cessation of antibiotics (16%). In our large cohort *only one infant* developed non-CONS-positive blood cultures in this time frame. This finding suggests the protocol allowed accurate identification of uninfected infants and thus a safe reduction of antimicrobial exposure. Protocol deviations were more common than expected (28%).

While still considered “gold standard” for the diagnosis of neonatal sepsis, blood cultures are known for their low sensitivity in VLBW infants [31]. Despite the relative infrequency of an early positive blood culture [32], antibiotics remain the most frequently prescribed medication in the NICU [1]. Multiple reports of the association of untoward consequences of prolonged antimicrobial use in the setting of a negative blood culture [6], [33], [34], raise significant concern and highlight the need for a means to safely limit treatment durations when possible. Many ancillary laboratory tests have been studied for sepsis diagnostic utility in neonates but exhibit significant limitations including a lack of wide availability and/or quick turnaround [35]. Two recent large retrospective studies suggested the diagnostic value of the WBC for the evaluation of sepsis in VLBW infants may be limited [20], [36]. Based on the reasons given for protocol deviation (Table 2), these data suggest WBC indices were by most commonly used to substantiate prolongation of antibiotic treatment in the setting of negative serial CRPs and a negative blood culture. Interestingly, the abnormal WBC indices cited as the reason to continue antibiotics in 64% of the Non-Comp group (Table 2) were also present during the entire sepsis evaluation period in 13% of the Comp group infants with safely discontinued antibiotics. This data supports the poor predictive value of abnormal WBC indices for neonatal sepsis [20]. Measurement of serum CRP is widely available, inexpensive, and can be used to identify uninfected infants and limit antimicrobial treatment [12]. In the present study, we have shown the utility of a computerized CRP-based sepsis evaluation protocol to safely reduce antimicrobial use in the VLBW preterm infant.

The use of CPOE with CDS has demonstrated utility and patient benefit [14]–[17]. This report is the first to describe the utility of CPOE with CDS to reduce the duration of early antimicrobial use without increasing the risk of negative outcomes in preterm VLBW infants.

Because hepatocytes are the primary source of CRP [37], we did not anticipate a greater incidence of falsely negative CRP in VLBWs with leukopenia but previous studies suggested this association [10], [12], [38]. On the other hand, in older populations with cancer and neutropenia, CRP levels rise with infection and may be higher in patients with neutropenia compared to those without [39], [40]. Although we found a weak correlation between ANC and CRP, we did not find a greater risk of falsely negative CRP values associated with low total or leukocyte counts of any type as compared to normal WBC with culture positive sepsis. Therefore have demonstrated that low WBC indices do not affect CRP values and no longer consider continuing empiric antibiotic therapy based on low WBC indices alone justifiable in almost all cases, if serial CRP values are negative.

There are noteworthy distinctions and limitations of our study. This study was retrospective and was not adequately powered to definitively determine the risk of common morbidities of prematurity. The lack of a comparison group treated without CRP protocol during the same time period does not allow us to claim overall reduction in antibiotic use. However, our results suggest that implementation of our protocol was associated with safe cessation of antimicrobial treatment in VLBW preterm infants unlikely to be infected. CPOE with CDS was implemented at our institution in 2003. Thus, at the time of CRP protocol initiation, all providers had adapted to the culture of CPOE, which may explain the relative high compliance with the protocol.

Any additional laboratory test can increase the risk for anemia in this vulnerable population. While we cannot exclude the possibility for increased anemia since introduction of the CDS/CRP-guided antibiotic use protocol, we do not expect a significant effect on anemia for the following reasons: 1) CRP values were almost always obtained at time of other routine laboratory tests (blood culture, CBC, bilirubin etc.) and 2) we only measured 2 CRP values within 48 hours followed by one additional value at 7 days of antibiotics in case initial CRP values suggested infection and a decision to treat was made. For better integration into provider workflow, the CRP protocol analyzed here used samples at initial diagnostic work-up. Given the lag period of CRP, elevated levels may not be evident until 12 hours after initiation of the inflammatory stimulus [13]. Since inflammatory markers with early kinetics such as IL-8 or IL-6 are not yet routinely available at our institution, we have now adopted a protocol that measures initial CRP values during rule-out of early onset sepsis during routine laboratory tests at 12 hours of age.

The computerized CRP protocol is used for determining the length of empiric antibiotic use in late-onset sepsis at our institution. We plan to evaluate the effectiveness and safety in this population as well but because of the greater heterogeneity of diagnoses and potential reasons to prolong antibiotic use (e.g. peri-operative), this project would fall outside the scope of this report. Another limitation is the definition of “confirmed positive blood culture”. The association of confirmed positive blood cultures and negative serial CRP values in our cohort was 12%. Others have reported negative serial CRP results in 36% of cases with gram-positive blood cultures and 8% with gram-negative blood cultures [26]. In that study, 14% of non-CONS single organism positive blood culture had negative serial CRP values similar to our results. After detailed chart review however, the description of clinical appearance in the majority of cases in our study was not consistent with sepsis and thus these cases might reasonably be considered asymptomatic positive blood cultures or contaminants. The rate of false-positive blood cultures in infants <12 weeks of age has been reported as high as 17% [41] and may be a particular problem in NICU patients who are frequently colonized with gram-negative bacteria [42].

While cut-off values may have to be lowered for extremely premature infants, we consider CRP a reliable marker for clinically significant positive blood cultures in NICU patients.

## Conclusions

We developed of a computerized CRP-based sepsis evaluation protocol for VLBW infants that was generally well accepted by health care providers, but WBC results influenced antibiotic duration decision-making despite CRP results. Persistently negative CRP values were rare in clinically probable non-CONS sepsis (1%), and do not occur more frequently in the presence of neutropenia.

## Supporting Information

Table S1 Shows the number and timing of medical and surgical necrotizing enterocolitis (NEC) cases and associated deaths by antibiotic group from cohort 1 (continued versus discontinued). Early NEC occurred more often in the antibiotics discontinued group but total number of NEC and total number of associated deaths were higher in the antibiotics continued group. The study was underpowered to test the effect of CRP protocol compliance on timing of NEC.
(DOCX)

## References

[1] Clark RH, Bloom BT, Spitzer AR, Gerstmann DR (2006) Reported medication use in the neonatal intensive care unit: data from a large national data set. Pediatrics 117: 1979–1987. 16740839 10.1542/peds.2005-1707

[2] Bizzarro MJ, Dembry LM, Baltimore RS, Gallagher PG (2008) Changing patterns in neonatal Escherichia coli sepsis and ampicillin resistance in the era of intrapartum antibiotic prophylaxis. Pediatrics 121: 689–696. 18381532 10.1542/peds.2007-2171

[3] Gerdes JS, Polin RA (1987) Sepsis screen in neonates with evaluation of plasma fibronectin. Pediatr Infect Dis J 6: 443–446. 3601490 10.1097/00006454-198705000-00005

[4] Hammerschlag MR, Klein JO, Herschel M, Chen FC, Fermin R (1977) Patterns of use of antibiotics in two newborn nurseries. N Engl J Med 296: 1268–1269. 859516 10.1056/NEJM197706022962206

[5] Philip AG, Hewitt JR (1980) Early diagnosis of neonatal sepsis. Pediatrics 65: 1036–1041. 7367117

[6] Cotten CM, Taylor S, Stoll B, Goldberg RN, Hansen NI, et al (2009) Prolonged duration of initial empirical antibiotic treatment is associated with increased rates of necrotizing enterocolitis and death for extremely low birth weight infants. Pediatrics 123: 58–66. 19117861 10.1542/peds.2007-3423 PMC2760222

[7] Bernard PA (1981) Freedom from ototoxicity in aminoglycoside treated neonates: a mistaken notion. Laryngoscope 91: 1985–1994. 7321720 10.1288/00005537-198112000-00001

[8] Stavroulaki P, Apostolopoulos N, Dinopoulou D, Vossinakis I, Tsakanikos M, et al (1999) Otoacoustic emissions – an approach for monitoring aminoglycoside induced ototoxicity in children. Int J Pediatr Otorhinolaryngol 50: 177–184. 10595663 10.1016/s0165-5876(99)00247-5

[9] Patel SJ, Saiman L (2010) Antibiotic resistance in neonatal intensive care unit pathogens: mechanisms, clinical impact, and prevention including antibiotic stewardship. Clin Perinatol 37: 547–563. 20813270 10.1016/j.clp.2010.06.004 PMC4440667

[10] Franz AR, Bauer K, Schalk A, Garland SM, Bowman ED, et al (2004) Measurement of interleukin 8 in combination with C-reactive protein reduced unnecessary antibiotic therapy in newborn infants: a multicenter, randomized, controlled trial. Pediatrics 114: 1–8. 15231900 10.1542/peds.114.1.1

[11] Franz AR, Steinbach G, Kron M, Pohlandt F (1999) Reduction of unnecessary antibiotic therapy in newborn infants using interleukin-8 and C-reactive protein as markers of bacterial infections. Pediatrics 104: 447–453. 10469768 10.1542/peds.104.3.447

[12] Benitz WE, Han MY, Madan A, Ramachandra P (1998) Serial serum C-reactive protein levels in the diagnosis of neonatal infection. Pediatrics 102: E41. 9755278 10.1542/peds.102.4.e41

[13] Weitkamp JH, Aschner JL (2005) Diagnostic use of C-reactive protein (CRP) in assessment of neonatal sepsis. Neoreviews 6: e508–515.

[14] Longhurst CA, Parast L, Sandborg CI, Widen E, Sullivan J, et al (2010) Decrease in hospital-wide mortality rate after implementation of a commercially sold computerized physician order entry system. Pediatrics 126: 14–21. 20439590 10.1542/peds.2009-3271

[15] Weitkamp JH, Ozdas A, LaFleur B, Potts AL (2008) Fluconazole prophylaxis for prevention of invasive fungal infections in targeted highest risk preterm infants limits drug exposure. J Perinatol 28: 405–411. 18185518 10.1038/sj.jp.7211914

[16] Taylor JA, Loan LA, Kamara J, Blackburn S, Whitney D (2008) Medication administration variances before and after implementation of computerized physician order entry in a neonatal intensive care unit. Pediatrics 121: 123–128. 18166565 10.1542/peds.2007-0919

[17] Ozdas A, Speroff T, Waitman LR, Ozbolt J, Butler J, et al (2006) Integrating “best of care” protocols into clinicians' workflow via care provider order entry: impact on quality-of-care indicators for acute myocardial infarction. J Am Med Inform Assoc 13: 188–196. 16357360 10.1197/jamia.M1656 PMC1447538

[18] Polin RA (2012) Management of neonates with suspected or proven early-onset bacterial sepsis. Pediatrics 129: 1006–1015. 22547779 10.1542/peds.2012-0541

[19] Fanaroff AA, Korones SB, Wright LL, Verter J, Poland RL, et al (1998) Incidence, presenting features, risk factors and significance of late onset septicemia in very low birth weight infants. The National Institute of Child Health and Human Development Neonatal Research Network. Pediatr Infect Dis J 17: 593–598. 9686724 10.1097/00006454-199807000-00004

[20] Hornik CP, Benjamin DK, Becker KC, Benjamin DK Jr, Li J, et al (2012) Use of the complete blood cell count in early-onset neonatal sepsis. Pediatr Infect Dis J 31: 799–802. 22531231 10.1097/INF.0b013e318256905c PMC3399972

[21] Manroe BL, Weinberg AG, Rosenfeld CR, Browne R (1979) The neonatal blood count in health and disease. I. Reference values for neutrophilic cells. J Pediatr 95: 89–98. 480023 10.1016/s0022-3476(79)80096-7

[22] Berrington JE, Barge D, Fenton AC, Cant AJ, Spickett GP (2005) Lymphocyte subsets in term and significantly preterm UK infants in the first year of life analysed by single platform flow cytometry. Clin Exp Immunol 140: 289–292. 15807853 10.1111/j.1365-2249.2005.02767.x PMC1809375

[23] Correa-Rocha R, Perez A, Lorente R, Ferrando-Martinez S, Leal M, et al (2012) Preterm neonates show marked leukopenia and lymphopenia that are associated with increased regulatory T-cell values and diminished IL-7. Pediatr Res 71: 590–597. 22398700 10.1038/pr.2012.6

[24] Christensen RD, Baer VL, Gordon PV, Henry E, Whitaker C, et al (2012) Reference ranges for lymphocyte counts of neonates: associations between abnormal counts and outcomes. Pediatrics 129: e1165–1172. 22508916 10.1542/peds.2011-2661

[25] Christensen RD, Jensen J, Maheshwari A, Henry E (2010) Reference ranges for blood concentrations of eosinophils and monocytes during the neonatal period defined from over 63 000 records in a multihospital health-care system. J Perinatol 30: 540–545. 20054336 10.1038/jp.2009.196

[26] Pourcyrous M, Bada HS, Korones SB, Baselski V, Wong SP (1993) Significance of serial C-reactive protein responses in neonatal infection and other disorders. Pediatrics 92: 431–435. 8361798

[27] Savica V, Santoro D, Mazzaglia G, Ciolino F, Monardo P, et al (2005) L-carnitine infusions may suppress serum C-reactive protein and improve nutritional status in maintenance hemodialysis patients. J Ren Nutr 15: 225–230. 15827896 10.1053/j.jrn.2004.10.002

[28] Duranay M, Akay H, Yilmaz FM, Senes M, Tekeli N, et al (2006) Effects of L-carnitine infusions on inflammatory and nutritional markers in haemodialysis patients. Nephrol Dial Transplant 21: 3211–3214. 16861734 10.1093/ndt/gfl356

[29] Hofer N, Muller W, Resch B (2011) Non-infectious conditions and gestational age influence C-reactive protein values in newborns during the first 3 days of life. Clin Chem Lab Med 49: 297–302. 21126207 10.1515/CCLM.2011.048

[30] Turner MA, Power S, Emmerson AJ (2004) Gestational age and the C reactive protein response. Arch Dis Child Fetal Neonatal Ed 89: F272–273. 15102735 10.1136/adc.2002.011288 PMC1721694

[31] Kaufman D, Fairchild KD (2004) Clinical microbiology of bacterial and fungal sepsis in very-low-birth-weight infants. Clin Microbiol Rev 17: 638–680. 15258097 10.1128/CMR.17.3.638-680.2004 PMC452555

[32] Stoll BJ, Hansen NI, Bell EF, Shankaran S, Laptook AR, et al (2010) Neonatal outcomes of extremely preterm infants from the NICHD Neonatal Research Network. Pediatrics 126: 443–456. 20732945 10.1542/peds.2009-2959 PMC2982806

[33] Kuppala VS, Meinzen-Derr J, Morrow AL, Schibler KR (2011) Prolonged initial empirical antibiotic treatment is associated with adverse outcomes in premature infants. J Pediatr 159: 720–725. 21784435 10.1016/j.jpeds.2011.05.033 PMC3193552

[34] Alexander VN, Northrup V, Bizzarro MJ (2011) Antibiotic exposure in the newborn intensive care unit and the risk of necrotizing enterocolitis. J Pediatr 159: 392–397. 21489560 10.1016/j.jpeds.2011.02.035 PMC3137655

[35] Benitz WE (2010) Adjunct laboratory tests in the diagnosis of early-onset neonatal sepsis. Clin Perinatol 37: 421–438. 20569816 10.1016/j.clp.2009.12.001

[36] Hornik CP, Benjamin DK, Becker KC, Benjamin DK Jr, Li J, et al (2012) Use of the complete blood cell count in late-onset neonatal sepsis. Pediatr Infect Dis J 31: 803–807. 22531232 10.1097/INF.0b013e31825691e4 PMC3399981

[37] Gabay C, Kushner I (1999) Acute-phase proteins and other systemic responses to inflammation. N Engl J Med 340: 448–454. 9971870 10.1056/NEJM199902113400607

[38] Sabel KG, Hanson LA (1974) The clinical usefulness of C-reactive protein (CRP) determinations in bacterial meningitis and septicemia in infancy. Acta Paediatr Scand 63: 381–388. 4209638 10.1111/j.1651-2227.1974.tb04814.x

[39] Povoa P, Souza-Dantas VC, Soares M, Salluh JF (2011) C-reactive protein in critically ill cancer patients with sepsis: influence of neutropenia. Crit Care 15: R129. 21595932 10.1186/cc10242 PMC3218995

[40] Santolaya ME, Cofre J, Beresi V (1994) C-reactive protein: a valuable aid for the management of febrile children with cancer and neutropenia. Clin Infect Dis 18: 589–595. 8038314 10.1093/clinids/18.4.589

[41] Norberg A, Christopher NC, Ramundo ML, Bower JR, Berman SA (2003) Contamination rates of blood cultures obtained by dedicated phlebotomy vs intravenous catheter. JAMA 289: 726–729. 12585951 10.1001/jama.289.6.726

[42] Graham PL 3rd, Begg MD, Larson E, Della-Latta P, Allen A, et al (2006) Risk factors for late onset gram-negative sepsis in low birth weight infants hospitalized in the neonatal intensive care unit. Pediatr Infect Dis J 25: 113–117. 16462286 10.1097/01.inf.0000199310.52875.10

