# Supplementary material for: Use of a Computerized C-Reactive Protein (CRP) Based Sepsis Evaluation in Very Low Birth Weight (VLBW) Infants: A Five-Year Experience
Source: PLoS One. 2013 Nov 11;8(11):e78602. doi: 10.1371/journal.pone.0078602 (PMC3823853; doi:10.1371/journal.pone.0078602)
Supplement: Table S1 — Shows the number and timing of medical and surgical necrotizing enterocolitis (NEC) cases and associated deaths by antibiotic group from cohort 1 (continued versus discontinued). Early NEC occurred more often in the antibiotics discontinued group but total number of NEC and total number of associated deaths were higher in the antibiotics continued group. The study was underpowered to test the effect of CRP protocol compliance on timing of NEC. (DOCX) [file pone.0078602.s001.docx]

**Table S1. Number and timing of medical and surgical necrotizing enterocolitis (NEC) cases and associated deaths by antibiotic group.**

|  | **Antibiotics Stopped** | | | | **Antibiotics Continued** | | | |
| --- | --- | --- | --- | --- | --- | --- | --- | --- |
| **Postpartum Day** | **Total NEC** | **Medical NEC** | **Surgical NEC** | **Deaths** | **Total NEC** | **Medical NEC** | **Surgical NEC** | **Deaths** |
| 0-10 | 6 | 3 | 3 | 1 | 0 | 0 | 0 | 0 |
| 11-20 | 4 | 3 | 1 | 0 | 6 | 6 | 0 | 1 |
| 21-30 | 4 | 3 | 1 | 0 | 9 | 7 | 2 | 3 |
| 31-40 | 4 | 2 | 2 | 0 | 6 | 4 | 2 | 0 |
| 41-60 | 3 | 2 | 1 | 0 | 6 | 5 | 1 | 1 |
| 61+ | 2 | 2 | 0 | 0 | 0 | 0 | 0 | 0 |
| Unknown | 1 | 1 | 0 | 0 | 0 | 0 | 0 | 0 |
| **Total** | **24 (7%)** | **16** | **8** | **1** | **27 (11%)** | **22** | **5** | **5** |
